# Prospective Study of the Assessment of Anxiety Symptoms after Minimally Invasive Lumbar Decompression

**DOI:** 10.1055/s-0044-1786727

**Published:** 2024-06-22

**Authors:** Roberto Maretti Meves, Pedro Cortat Couri, Eliane Antonioli, Albert Ofenhejm Gotfryd

**Affiliations:** 1 Faculdade Israelita de Ciências da Saúde Albert Einstein, São Paulo, SP, Brasil; 2 Hospital Israelita Albert Einstein, São Paulo, SP, Brasil; 3 Pós-Graduação em Ortopedia Multiprofissional, Hospital Israelita Albert Einstein, São Paulo, SP, Brasil; 4 Treinamento de Cirurgiões de Coluna, Hospital Israelita Albert Einstein, São Paulo, SP, Brasil

**Keywords:** anxiety, depression, estenosis, minimally invasive surgical procedures, spine

## Abstract

**Objective** To analyze associations between anxiety and postsurgical clinical outcomes in patients who underwent minimally invasive lumbar decompression surgery in addition to comparing symptoms of anxiety and depression before and after surgery.

**Methods** This prospective cohort study of patients who underwent minimally invasive lumbar decompression surgery. Clinical outcomes were measured before and 6 months after surgery using the Visual Analog Scale (VAS), Global Perceived Effect of Change (GPE), Hospital Anxiety and Depression Scale (HADS), and Oswestry Disability Index (ODI). Based on the presurgical anxiety score, patients were categorized into anxious and non-anxious patients, and the outcomes were compared.

**Results** The patients of both groups obtained similar results concerning the clinical outcomes evaluated. Preoperative HADS scores decreased significantly 6 months after surgery in both anxiety (8.70 ± 3.48 vs. 5.75 ± 3.91) and depression (6.95 ± 3.54 vs. 5.50 ± 2.99). The VAS scale for the back (-2.8 ± 3.64) and legs (-5.5 ± 3.5) showed a reduction in pain.

**Conclusion** Minimally invasive lumbar decompression surgery promoted clinical and functional improvement, not being affected by preoperative anxiety symptoms. Mental health indicators showed a significant reduction in symptoms 6 months after surgery.

## Introduction

The main reason for surgery on the spine is lumbar neural compression, which promotes the sensation of paresthesia, pain, loss of sensitivity, and strength in the lower limbs. 1 The most common reasons for lumbar neural compression are spinal canal stenosis and herniated discs. In the failure of conservative treatment or the presence of motor neurological damage, operative treatment should be indicated, with the gold standard being neural decompression. The procedure can be performed openly or microsurgically, with tubular retractors or endoscopy. 2 3 Minimally invasive lumbar decompression is a relatively new technique that represents an alternative to open decompression. 4 The minimally invasive technique demonstrates similar effectiveness to open laminectomy but with lower blood loss, shorter hospital stays, and lower incidence of reoperation. 5 6

Previous studies have shown an association between symptoms of anxiety, depression, and chronic low back pain. 7 However, the relationship between anxiety symptoms and surgical outcomes in individuals with radiculopathy is not well-established in the literature. Thus, the present study investigates the association between surgical outcomes and the presence of preoperative anxiety symptoms, in addition to the effects of surgery on patients' mental health over time.

The objectives were: Primary - to evaluate the association between anxiety symptoms and clinical and functional outcomes of patients undergoing minimally invasive lumbar decompression. Secondary - compare anxiety symptoms before and after minimally invasive lumbar decompression surgery.

## Methodology

This is a prospective cohort study with 44 patients, with follow-up for 6 months after surgery between January 2020 and July 2022. All procedures were performed at the Hospital Israelita Albert Einstein, a private quaternary hospital, a reference in spine surgery, located in the city of São Paulo. The study was approved by the institution's Ethics Committee (CAAE: 94868618.7.0000.0071).

**Inclusion criteria:** Individuals who had symptoms of lumbar neural compression, radiculopathy or neurogenic lameness, single-level lumbar neural compression, with failure of conservative treatment for at least 6 weeks (or less, in the presence of acute motor damage with a motor force of less than or equal to 3), underwent single-level minimally invasive lumbar decompression.

**Exclusion criteria:** Open surgeries, placement of implants in the spine, reoperations, complications, or the absence of response to questionnaires after 6 months of surgery.

The collected data was sent and stored in the REDCap software (Vanderbilt University, Nashville, TN, USA 8 9 ), at the time before and 6 months after surgery. The preoperative questionnaires were collected shortly after the informed consent form (ICF) was completed.

### Operative Technique

The patients were positioned in the genupectoral position without needing bladder probing. The fluoroscopic image in profile incidence confirmed the anatomical level, with the needle positioned at the height of the intervertebral disc. After placing sterile drapes, a paramedian incision was made, located 1 cm lateral to the midline and 14 mm long, corresponding to the size of the tubular retractor used. After opening the fascia, the musculature was progressively dilated with sequential cannulas until the placement of the 14-mm-diameter closed tube. The tube length varied according to each patient, between 50 and 90 mm deep. The tube was attached to the operating table using a mechanical arm, and from that moment on, the surgery was performed using microscopy (Pentero microscope, Zeiss, Germany). Neural decompression was performed according to the needs of each case. It may include microdiscectomy for cases of a herniated disc, foraminotomy for foraminal stenosis, decompression of lateral recess stenosis, or *over-the-top decompression* for central stenosis. After that, the wound was closed with continuous intradermal suture and simple dressing. Drains were not inserted. Ambulation was authorized on the same day of surgery, and discharge occurred the day after surgery. The surgeries were performed by a senior physician who specialized in minimally invasive spine surgery.

### Collected Outcomes

***Visual Analog Pain Scale*(VAS)** : 10 The visual pain scale describes the intensity of the patient's leg and back pain-related pain. The score can range from 0 (non-existent pain) to 10 (pain with extreme intensity)

***Global Perceived Effect of Change*(GPE)** (self-perceived improvement) 11 : Scale designed to quantify patient improvement or deterioration over time, usually to determine the effect of an intervention or to chart the clinical course of a condition.

***Hospital Anxiety and Depression Scale*(HADS)** : 12 The questionnaire consists of 14 questions. Each of its items can be scored from 0 to 3, composing a maximum score of 21 points for each scale.

***Oswestry Disability Index*(ODI)** Version 2.0: 13 This version was adapted for Brazil to measure functional disability in individuals with low back pain. It has 10 scales of 6 points each. The total ODI score ranges from 0 (no disability) to 100 (maximum disability).

### Categorization of patients with and without anxiety symptoms

Patients were stratified into two groups depending on the score at the presurgical moment of anxiety (HADS). The chosen cutoff point (≥ 9) in the anxiety score was based on the description of Zigmond and Snaith 14 on the interpretation of results.

**Group 1: non-anxious patients.** Hospital Anxiety and Depresseion Scale subscale anxiety collected presurgically < 9 points
**Group 2: anxious patients.** Hospital Anxiety and Depression Scale subscale anxiety collected presurgically ≥ 9 points

### Statistical analysis

To evaluate the different associations between the groups, the variations of scores (depression HADS, VAS, GPE) and final scores in the groups were calculated separately. The data were submitted to Shapiro-Wilk normality tests. Subsequently, non-parametric statistical tests were applied to measure the difference in postoperative outcomes between patients' “cases” versus “non-cases” of anxiety. Categorical variables were followed by the percentage that each category occupies and continuous variables by the standard deviation. Nonparametric statistical tests were performed to compare the initial and postoperative scores. The statistical tests applied for continuous variables were Mann-Whitney and, for categorical variables, the Pearson chi-square test. Subsequently, an analysis was performed comparing the evolution of anxious patients with that of non-anxious patients. This comparison was made by evaluating the variation of scores (final score–initial score) and comparing them between the groups.

## Results

The sample consisted of 44 patients, 22 males and 22 females, with a mean age of 42.5 years (20–70 years). The mean body mass index (BMI) of the sample was 27.1 kg/m ^2^ . Most of the patients have completed higher education. Table 1 presents the patients' demographic data stratified in relation to the HADS score at the preoperative time, with 20 patients in the non-anxious group and 24 patients in the anxious group. Demographic data, such as BMI, age, height, weight, education, and sex, are similar between groups.

**Table 1:** Initial characteristics of the studied population

		Group	
	All	Non-anxious	Anxious
	(n = 44)	(n = 20)	(n = 24)
Gender			
Male (%)	22	12	10
Female (%)	22	8	14
Schooling			
Undergraduate degree	33	17	16
Unfinished undergraduate degree	4	0	4
High school degree	5	2	3
Middle school degree	1	0	1
Technical degree	1	1	0
Age	42.5 ± 10.4	45.6 ± 10.4	39.9 ± 9.76
BMI (Kg/m2)	27.1 ± 3.8	26.8 ± 3.52	27.3 ± 4.09
Height (m)	1.7 ± 0.1	1.71 ± 9.13	1.71 ± 12.1
Weight (Kg)	79.4 ± 14.4	79.3 ± 16	79.6 ± 13.2

Abbreviation: BMI, body mass index.

Age, body mass index (BMI), height, and weight presented with ± standard deviation (±95% confidence interval).

In the comparison between preoperative clinical scores and those 6 months after the procedure, improvement was observed in all scores ( *p* < 0.05), regardless of the group. Clinical improvement was observed by the increase in self-perceived improvement (GPE), reduction in leg and back pain scores, and HADS scores for both subscales (depression and anxiety), according to Table 2 .

**Table 2:** Sample data after 6 months of surgery and mean variation

		Preoperative				6 months after surgery				Score variation 6 months	
	Non-anxious		Anxious	p	Non-anxious		Anxious	p	Non-anxious		Anxious
**HADS**											
**Anxiety**	5.65 ± 2.06		11.3 ± 2.07	<0.001*	3.9 (±3.42)		7.29 (± 3.66)	0.003	-1.75 (±3.61)		-3.96 (±3.84)
**Depression**	4.7 ± 2.13		8.83 ± 3.41	<0.001*	4.1 (±1.83)		6.67 (±3.29)	0.003	-0.6 (±2.76)		-2.17 (±3.06)
**VAS**											
**Back**	4.6 ± 3.22		5.92 ± 2.7	0.16	2.45 (±3.22)		2.58 (±2.41)	0.562	-2.15 (±3.9)		-3.33 (±3.41)
**Legs**	6.6 ± 2.89		7.72 ± 2.4	0.44	1.8 (±2.78)		1.62 (±2.24)	0.919	-4.8 (±4.21)		-6.08 (±2.73)
**GPE**	-0.6 ± 3.55		-0.38 ± 3.51	0.73	2.6 (±2.89)		2.33 (±2.56)	0.457	3.2 (±5.4)		2.71 (±4.36)
**ODI**	36.5 ± 20.8		52.8 ± 22.9	0.02*	10.4 (±10.4)		13.8 (±10.9)	0.29	-26.1 (±21.4)		-37.9 (±21.9)

Abbreviations: HADS, Hospital Anxiety and Depression Scale; VAS, Visual Analogue Scale; GPE, Global Perceived Effect of Change; ODI, Oswestry Disability Index.

*P* -value obtained using the Mann-Whitney test.

Six months after surgery, anxious patients maintained statistically worse mental health scores compared to non-anxious patients. However, the score reduction in the 6 months was higher in anxious patients. That is, anxious patients maintained lower mental health scores after surgery compared to non-anxious patients but obtained a more significant reduction in points (-3.96 vs -1.75); this pattern is repeated in depression scores.

Another finding was the different variations of the ODI score between the groups. While the anxious group had a drop of 37.9 points, on average, the non-anxious group had a reduction of 26.1 points. In this sense, the anxious group had a 74% reduction in the average ODI score, and the non-anxious group had 71.5%.

The other scores showed similar variation between the groups. Except for the GPE score, all scores showed more significant variation between preoperative and postoperative moments in the anxious group. Regarding the back VAS score, there was a reduction of 47% in not anxious and 56% in anxious patients. The leg VAS, in turn, had a reduction of 79% in anxious and 72.8% in non-anxious patients.

Table 3 presents the evolution of patients without considering the classification of anxiety and depression, showing significant variation in mental health indicators. After 6 months of the procedure, there was a reduction in the HADS scores in both anxiety and depression subscales and in the other scores. The GPE and ODI scores increased in the sample, respectively, demonstrating increased self-perception of improvement and functionality.

**Table 3:** Symptoms and function preoperative and 6 months after surgery

	Preoperative		6 months after surgery		
					*p*
**HADS**					
Anxiety	8.7 (±3.48)		5.91 (±4.01)		< 0.001
Depression	6.95 (±3.54)		5.6 (±3.03)		0.005
**VAS**					
Back	5.32 (±3.03)		2.52 (±2.70)		< 0.001
Legs	7.2 (±2.66)		1.70 (±2.47)		< 0.001
**GPE**	-0.48 (±3.48)		2.45 (±2.69)		< 0.001
**ODI**	45.4 (±23.2)		12.2 (±10.70)		< 0.001

Abbreviations: HADS, *Hospital Anxiety and Depression Scale* ; VAS, Visual Analogue Scale; GPE, Global Perceived Effect of Change; ODI, Oswestry Disability Index.

*P* -value obtained by Wilcoxon test.

**Fig. 1:**
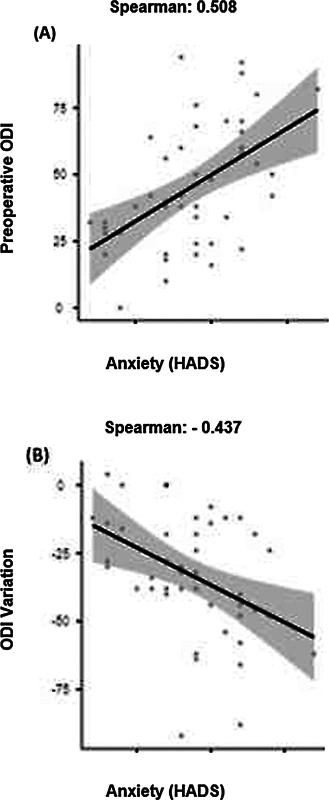
Anxiety scatter plot x ODI. Spearman rank coefficient.

A positive correlation was observed between preoperative ODI ( *p* < 0.001) and preoperative anxiety ( Fig. 1A ); that is, patients with higher anxiety scores reported greater dysfunction function at the preoperative time. In addition, a negative correlation was observed between preoperative anxiety and ODI score variation, that is, patients with initially higher anxiety scores reported greater functional gain after surgery.

## Discussion

Spine surgeries have evolved in recent years, and techniques considered less invasive become increasingly used. 15 In the present study, microtubular and endoscopic surgery was used, which proposes little tissue aggression and an early return to normal preoperative activities. 16 This approach promoted pain improvement, self-perceived improvement, anxiety, and depression symptoms 6 months after surgery.

The age of the patients in this study corresponds to that reported in other studies, with the mean age of patients with neural compression being 35 to 55 years. 17

Previous studies correlate chronic low back pain with worsening mental health. 18 19 However, the impact of anxiety symptoms on the clinical and functional outcomes of patients with neural compression undergoing minimally invasive lumbar decompression is not well established in the literature. Specific studies on the subject suggest that anxiety symptoms do not modify the prognosis of patients. 20 However, the initial hypothesis of the present study was that anxious patients would have worse clinical and functional outcomes. Contrary to the initial hypothesis, and in agreement with previous studies, it was observed that more anxious patients had similar clinical outcomes to those of non-anxious patients. However, studies on the effects of anxiety on clinical outcomes should continue since it is a comorbidity, and these, in turn, are obstacles in the postoperative treatment of patients.

Regarding the modulation of the anxiety score in patients after surgical treatment, the data obtained suggest that, in individuals adequately selected for neural compression surgery, anxiety symptoms would be caused by pain, not originate it. Six months after surgery, a significant reduction in anxiety symptoms was observed, accompanied by an improvement in pain and quality of life scores.

The mean variation of the functionality scale during the 6 months was significant (33.2 points), similar to the variation of surgical decompression described in the systematic review by Ma et al. 21 (28.1–34.5).

Concerning the association tables analyzed, the association between dysfunction and preoperative anxiety follows that analyzed in previous studies, which suggests worse anxiety symptoms in patients with more intense lumbar compression symptoms. Simultaneously, the positive correlation between functional gain and preoperative anxiety demonstrates that patients with more anxiety symptoms had more significant benefits from surgery in terms of functionality.

The main limitations of this study are its small sample size, its limitation to a private hospital center, and its potential inability to represent the reality of other services. The use of the HADS questionnaire for anxiety screening can be considered limited due to the lack of face-to-face evaluation by a psychologist. Due to the absence of evaluation of psychiatric comorbidities in patients and their medication use, it was not possible to evaluate possible effects between psychiatric comorbidities, medication use, and the development of anxiety symptoms in patients after surgery. However, the consistency of the data suggests that the presence of neuropathic pain and dysfunction in individuals with lumbar neurological compression promoted the appearance of anxiety symptoms. The factors that corroborate this hypothesis are the initial correlation between dysfunction and anxiety, followed by a decrease in both due to the therapeutic effect of surgery on dysfunction, consequently reducing anxiety.

## Conclusions

Minimally invasive lumbar decompression surgery promoted clinical and functional improvement, not being affected by preoperative anxiety symptoms. These, in turn, improved 6 months after surgery.
