# Supplementary material for: Prospective Study of the Assessment of Anxiety Symptoms after Minimally Invasive Lumbar Decompression
Source: Rev Bras Ortop (Sao Paulo). 2024 Jun 22;59(3):e372–7. doi: 10.1055/s-0044-1786727 (PMC11193580; doi:10.1055/s-0044-1786727)
Supplement: Related texts

# Research article: Estudo prospectivo da avaliação dos sintomas de ansiedade após descompressão lombar minimamente invasiva

**Keywords:** ansiedade, coluna vertebral, depressão, estenose, procedimentos cirúrgicos minimamente invasivos

## Abstract

**Objetivo** Analisar associações entre ansiedade e desfecho clínico pós-cirúrgico em pacientes que realizaram a cirurgia de descompressão lombar minimamente invasiva, além de comparar sintomas de ansiedade e depressão antes e após a cirurgia.

**Métodos** Trata-se de um estudo de coorte prospectiva de pacientes que realizaram a cirurgia de descompressão lombar minimamente invasiva. Os desfechos clínicos foram mensurados antes e 6 meses após a cirurgia, utilizando a escala visual analógica de dor (EVA); *Global Perceived Effect of Change* (GPE); *Hospital Anxiety and Depression Scale* (HADS) e o *Oswestry Disability Index* (ODI). A partir do escore de ansiedade pré-cirúrgico, os pacientes foram categorizados em pacientes ansiosos e não ansiosos e os desfechos comparados.

**Resultados** Os pacientes de ambos os grupos obtiveram resultados similares em relação aos desfechos clínicos avaliados. Os escores HADS pré-operatório diminuíram significantemente 6 meses após a cirurgia, tanto em ansiedade (8,70 ± 3,48 vs. 5,75 ± 3,91) quanto em depressão (6,95 ± 3,54 vs. 5,50 ± 2,99). Houve redução da dor na escala EVA das costas (-2,8 ± 3,64) e das pernas (-5,5 ± 3,5).

**Conclusão** A cirurgia de descompressão lombar minimamente invasiva promoveu melhora clínica e funcional, não sendo afetada por sintomas pré-operatórios de ansiedade. Os indicadores de saúde mental evidenciaram redução significativa dos sintomas 6 meses após a cirurgia.

## Introdução

O principal motivo de cirurgia na coluna vertebral é compressão neural lombar, que promove a sensação de parestesia, dor, perda de sensibilidade e força nos membros inferiores. 1 Os motivos mais comuns de compressão neural lombar são estenose do canal vertebral e hérnia de disco. Na falha do tratamento conservador ou na presença de dano neurológico motor, o tratamento operatório deve ser indicado, sendo padrão-ouro a descompressão neural. O procedimento pode ser realizado de forma aberta ou microcirúrgica, com retratores tubulares ou por endoscopia. 2 3 A descompressão lombar minimamente invasiva é uma técnica relativamente nova, que representa uma alternativa à descompressão aberta. 4 Quando comparada à laminectomia aberta, a técnica minimamente invasiva demonstra efetividade similar, porém com menor perda de sangue, menor tempo de internação hospitalar e menor incidência de reoperação. 5 6

Estudos prévios mostraram associação entre os sintomas de ansiedade, depressão e dor lombar crônica. 7 Entretanto, a relação entre sintomas de ansiedade e resultados cirúrgicos em indivíduos com radiculopatia não é bem estabelecida na literatura. Dessa forma, o presente estudo investiga a associação entre os desfechos cirúrgicos e a presença de sintomas de ansiedade pré-operatórios, além dos efeitos da cirurgia na saúde mental dos pacientes ao longo do tempo.

Os objetivos foram: primário - avaliar a associação entre sintomas de ansiedade e resultados clínicos e funcionais de pacientes submetidos a descompressão lombar minimamente invasiva. Secundário - comparar sintomas de ansiedade antes e após a cirurgia de descompressão lombar minimamente invasiva.

## Metodologia

Trata-se de estudo tipo coorte prospectiva com 44 pacientes, com acompanhamento por 6 meses após a cirurgia, no período entre janeiro de 2020 a julho de 2022. Todos os procedimentos foram realizados no Hospital Israelita Albert Einstein, hospital quaternário privado, referência em cirurgia de coluna, localizado na cidade de São Paulo. O estudo foi aprovado pelo Comitê de Ética da Instituição (CAAE: 94868618.7.0000.0071).

**Critérios de inclusão:** indivíduos que apresentavam sintomas de compressão neural lombar, radiculopatia ou claudicação neurogênica, compressão neural lombar em único nível, com falha do tratamento conservador mínimo por 6 semanas (ou menos, na presença de dano motor agudo com força motora menor ou igual a 3), submetidos a descompressão lombar minimamente invasiva em único nível.

**Critérios de exclusão:** cirurgias abertas, colocação de implantes na coluna, reoperações, intercorrências ou a ausência de resposta aos questionários após 6 meses da cirurgia.

Os dados coletados foram enviados e armazenados no software REDCap (Vanderbilt University, Nashville, TN, EUA 8 9 ) no momento prévio à cirurgia e 6 meses após a cirurgia. Os questionários pré-operatórios foram coletados logo após o preenchimento do termo de consentimento livre e esclarecido (TCLE).

## Técnica operatória

Os pacientes foram posicionados em posição genupeitoral sem necessidade de sondagem vesical. O nível anatômico foi confirmado por imagem fluoroscópica na incidência perfil, com agulha posicionada na altura do disco intervertebral. Após colocação de campos estéreis, realizou-se incisão paramediana localizada 1 cm lateral à linha média com 14 mm de extensão, correspondente ao tamanho do retrator tubular utilizado. Após abertura da fáscia, a musculatura foi progressivamente dilatada com cânulas sequenciais até a colocação do tubo fechado de 14 mm de diâmetro. O comprimento do tubo variou de acordo com cada paciente, entre 50 e 90 mm de profundidade. O tubo foi acoplado à mesa cirúrgica por meio de um braço mecânico e, desse momento em diante, a cirurgia foi realizada com auxílio de microscopia (Microscópio Pentero, Carl Zeiss AG, Oberkochen, Alemanha). A descompressão neural foi realizada de acordo com a necessidade de cada caso, podendo compreender: microdiscectomia para casos de hérnia de disco, foraminotomia para estenose foraminal, descompressão de estenose de recesso lateral ou *descompressão over-the-top* para estenose central. Após isso, a ferida foi fechada com sutura contínua intradérmica e curativo simples. Drenos não foram inseridos. A marcha foi autorizada no mesmo dia da cirurgia e a alta ocorreu no dia seguinte à mesma. As cirurgias foram realizadas por médico sênior (AG) especializado na cirurgia de coluna minimamente invasiva.

## Desfechos coletados

***Escala visual analógica da dor*(EVA)**:10 a escala visual de dor descreve a intensidade da dor do paciente, relacionado a dor nas pernas, e outro a dor nas costas. O escore pode variar de 0 (dor inexistente) a 10 (dor com intensidade extrema)

***Global Perceived Effect of Change*(GPE)** (autopercepção de melhora) 11 : escala projetada para quantificar a melhora ou deterioração do paciente ao longo do tempo, geralmente para determinar o efeito de uma intervenção ou para traçar o curso clínico de uma condição.

***Hospital Anxiety and Depression Scale*(HADS)** : 12 O questionário consiste em 14 perguntas. Cada um dos seus itens pode ser pontuado de 0 a 3, compondo uma pontuação máxima de 21 pontos para cada escala.

***Oswestry Disability Index*(ODI)** Versão 2.0 13 : adaptada para o Brasil para medir incapacidade funcional em indivíduos com lombalgia, com 10 escalas de 6 pontos cada. A pontuação total do ODI varia de 0 (sem deficiência) a 100 (incapacidade máxima).

## Categorização dos pacientes com e sem sintomas de ansiedade

Os pacientes foram estratificados em dois grupos dependendo do escore no momento pré-cirúrgico de ansiedade (HADS). O ponto de corte escolhido (≥ 9) no escore de ansiedade foi baseado na descrição de Zigmond e Snaith 14 sobre interpretação de resultados.

**Grupo 1: pacientes não-ansiosos.** Subescala HADS ansiedade coletada no momento pré-cirúrgico < 9 pontos
**Grupo: pacientes ansiosos.** Subescala HADS ansiedade coletada no momento pré cirúrgico ≥ 9 pontos

## Análise estatística

Para avaliar as diferentes associações entre os grupos foram calculadas as variações de escores (HADS depressão, EVA, GPE) e escores finais nos grupos separadamente. Os dados foram submetidos a testes de normalidade Shapiro-Wilk. Posteriormente, foram aplicados testes estatísticos não paramétricos, com o intuito de medir a diferença de resultados pós-operatórios entre os pacientes “casos” versus “não-casos” de ansiedade. As variáveis categóricas foram acompanhadas pela porcentagem que cada categoria ocupa, e as variáveis contínuas pelo desvio padrão. Foram realizados testes estatísticos não paramétricos para comparar os escores iniciais com os escores pós-operatórios. Os testes estatísticos aplicados para variáveis contínuas foram Mann-Whitney e para variáveis categóricas o teste de Pearson qui-quadrado. Posteriormente, realizou-se análise comparando a evolução dos pacientes ansiosos com a dos não ansiosos. Esta comparação foi feita avaliando a variação de escores (escore final–escore inicial) e comparando-as entre os grupos.

## Resultados

A amostra consistiu em 44 pacientes, 22 do gênero masculino e 22 do gênero feminino, com média de idade de 42,5 anos (20–70 anos). O índice de massa corporal (IMC) médio da amostra foi 27,1 Kg/m ^2^ . Os pacientes, em sua maioria, completaram o ensino superior. A Tabela 1 apresenta os dados demográficos dos pacientes foram estratificados em relação ao escore HADS no momento pré-operatório, contendo 20 pacientes no grupo não ansiosos e 24 pacientes no grupo ansiosos. Os dados demográficos IMC, idade, altura, peso, escolaridade e sexo são similares entre os grupos.

**Tabela 1:** Características iniciais da população estudada

		GRUPO	
	Todos	Não ansiosos	Ansiosos
	(n = 44)	(n = 20)	(n = 24)
Gênero			
Masculino (%)	22	12	10
Feminino (%)	22	8	14
Escolaridade			
Superior completo	33	17	16
Superior incompleto	4	0	4
Médio completo	5	2	3
Fundamental completo	1	0	1
Ensino técnico	1	1	0
Idade	42,5 ± 10,4	45,6 ± 10,4	39,9 ± 9,76
IMC (Kg/m2)	27,1 ± 3,8	26,8 ± 3,52	27,3 ± 4,09
Altura (m)	1,7 ± 0,1	1,71 ± 9,13	1,71 ± 12,1
Peso (Kg)	79,4 ± 14,4	79,3 ± 16	79,6 ± 13,2

Abreviatura: IMC, índice de massa corporal.

Idade, índice de massa corporal (IMC), altura e peso apresentados com ±**** desvio padrão (±95% intervalo de confiança).

Na comparação entre os escores clínicos no momento pré-operatório e 6 meses após o procedimento, observou-se melhora em todos os eles ( *p* < 0,05), independente do grupo. A melhora clínica foi observada pelo aumento na autopercepção de melhora (GPE), redução dos escores de dor na perna e nas costas e escores HADS para ambas subescalas (depressão e ansiedade), de acordo com a Tabela 2 .

**Tabela 2:** Dados da amostra após 6 meses da cirurgia e variação de médias

		Pré-operatório				6 meses após a cirurgia				Variação de escore 6 meses	
	Não Ansiosos		Ansiosos	p	Não Ansiosos		Ansiosos	p	Não Ansiosos		Ansiosos
**HADS**											
**Ansiedade**	5,65 ± 2,06		11,3 ± 2,07	<0,001*	3,9 (±3,42)		7,29 (±3,66)	0,003	-1,75 (±3,61)		-3,96 (±3,84)
**Depressão**	4,7 ± 2,13		8,83 ± 3,41	<0,001*	4,1 (±1,83)		6,67 (±3,29)	0,003	-0,6 (±2,76)		-2,17 (±3,06)
**EVA**											
**Costas**	4,6 ± 3,22		5,92 ± 2,7	0,16	2,45 (±3,22)		2,58 (±2,41)	0,562	-2,15 (±3,9)		-3,33 (±3,41)
**Pernas**	6,6 ± 2,89		7,72 ± 2,4	0,44	1,8 (±2,78)		1,62 (±2,24)	0,919	-4,8 (±4,21)		-6,08 (±2,73)
**GPE**	-0,6 ± 3,55		-0,38 ± 3,51	0,73	2,6 (±2,89)		2,33 (± 2,56)	0,457	3,2 (±5,4)		2,71 (±4,36)
**ODI**	36,5 ± 20,8		52,8 ± 22,9	0,02*	10,4 (±10,4)		13,8 (±10,9)	0,29	-26,1 (±21,4)		-37,9 (±21,9)

Abreviaturas: HADS, Hospital Anxiety and Depression Scale; EVA, Escala Visual Análoga; GPE, Global Perceived Effect of Change; ODI, Oswestry Disability Index.

Valor de *p* obtido por meio do teste de Mann-Whitney.

Após 6 meses da cirurgia, os pacientes ansiosos mantiveram escores estatisticamente piores de saúde mental em comparação com os pacientes não ansiosos. Porém, a redução nos escores no período de 6 meses foi superior em pacientes ansiosos. Ou seja, pacientes ansiosos mantiveram escores de saúde mental inferiores após a cirurgia, em comparação com pacientes não ansiosos, mas obtiveram maior redução de pontos (-3,96 vs -1,75). Este padrão se repete nos escores de depressão.

Outro achado foi a variação diferente do escore ODI entre os grupos. Enquanto o grupo de pacientes ansiosos teve uma queda de 37,9 pontos em média, o grupo de pacientes não ansiosos obteve redução de 26,1 pontos. Nesse sentido, o grupo ansiosos teve uma redução de 74% do escore ODI médio e o grupo não ansiosos teve redução de 71,5%.

Os demais escores apresentaram variação de forma similar entre os grupos. Com exceção do escore GPE, todos os escores demonstraram variação entre os momentos pré e pós-operatório maior no grupo ansiosos. Em relação ao escore EVA das costas houve uma redução de 47% em pacientes não ansiosos e de 56% em pacientes ansiosos. A EVA das pernas, por sua vez, teve uma redução de 79% em pacientes ansiosos e de 72,8% em pacientes não ansiosos.

A Tabela 3 apresenta a evolução dos pacientes sem considerar a classificação de ansiedade e depressão, mostrando variação significativa dos indicadores de saúde mental. Após 6 meses do procedimento, observa-se redução dos escores HADS em ambas as subescalas, de ansiedade e depressão, e nos demais escores. Os escores GPE e ODI aumentaram na amostra, demonstrando, respectivamente, aumento da autopercepção de melhora e funcionalidade.

**Tabela 3:** Sintomas e função no pré-operatório e 6 meses após a cirurgia

	Pré-operatórios		6 meses após a cirurgia		
					*p*
**HADS**					
Ansiedade	8,7 (±3,48)		5,91 (±4,01)		< 0,001
Depressão	6,95 (±3,54)		5,6 (±3,03)		0,005
**EVA**					
Costas	5,32 (±3,03)		2,52 (±2,70)		< 0,001
Pernas	7,2 (±2,66)		1,70 (±2,47)		< 0,001
**GPE**	-0,48 (±3,48)		2,45 (± 2,69)		< 0,001
**ODI**	45,4 (± 23,2)		12,2 (±10,70)		< 0,001

Abreviaturas: HADS, *Hospital Anxiety and Depression Scale* ; EVA, Escala Visual Análoga; GPE, Global Perceived Effect of Change; ODI, Oswestry Disability Index.

Valor de *p* obtido por meio do teste de Wilcoxon.

Observou-se correlação positiva entre o ODI no momento pré-operatório ( *p* < 0,001) e ansiedade no momento pré-operatório ( Fig. 1A ), ou seja, pacientes com maiores escores de ansiedade relataram maior disfunção função no momento pré-operatório. Além disso, observou-se correlação negativa entre ansiedade no momento pré-operatório e variação do escore ODI, ou seja, pacientes com escores de ansiedade inicialmente maiores relataram maior ganho de funcionalidade após a cirurgia.

**Fig. 1:** Gráfico de dispersão ansiedade x ODI. Coeficiente de postos de Spearman.

## Discussão

As cirurgias de coluna têm evoluído nos últimos anos e técnicas consideradas de menor invasão tornam-se cada vez mais utilizadas. 15 No presente estudo, utilizou-se a técnica da cirurgia microtubular e endoscópica, que propõe pouca agressão tecidual e retorno precoce às atividades habituais pré-operatórias. 16 Tal abordagem promoveu melhora da dor, autopercepção de melhora, e melhora dos sintomas de ansiedade e depressão após 6 meses da cirurgia.

A idade dos pacientes deste estudo corresponde ao reportado em outros estudos, com idade média dos pacientes com compressão neural de 35 a 55 anos. 17

Estudos prévios correlacionam dor lombar crônica com piora da saúde mental. 18 19 Entretanto, o impacto de sintomas de ansiedade nos resultados clínicos e funcionais de pacientes com compressão neural submetidos a descompressão lombar minimamente invasiva não é bem estabelecido na literatura. Estudos específicos sobre o assunto sugerem que sintomas de ansiedade não modificam o prognóstico dos pacientes. 20 Entretanto, a hipótese inicial do presente estudo foi que pacientes ansiosos teriam piores resultados clínicos e funcionais. Contrariamente à hipótese inicial, e em concordância com estudos prévios, observou-se que pacientes mais ansiosos tiveram desfechos clínicos similares aos dos pacientes não ansiosos. Todavia, estudos sobre os efeitos da ansiedade sobre os desfechos clínicos devem prosseguir, dado que se trata de uma comorbidade e estas, por sua vez, são empecilhos no tratamento pós-operatório dos pacientes.

Em relação à modulação do escore de ansiedade em pacientes após o tratamento cirúrgico, os dados obtidos sugerem que, em indivíduos adequadamente selecionados para cirurgia por compressão neural, sintomas de ansiedade seriam causados pela dor, não sendo origem dela. Uma vez que, 6 meses após a cirurgia, observou-se redução significativa dos sintomas de ansiedade, acompanhado pela melhora da dor e dos escores de qualidade de vida.

Observou-se que a média da variação da escala de funcionalidade durante os 6 meses foi significativa (33,2 pontos), similar à variação da descompressão cirúrgica descrita na revisão sistemática de Ma et al. 21 (28,1–34,5).

No que tange as tabelas de associação analisadas, a associação entre disfunção e ansiedade no momento pré-operatório segue o analisado em estudos prévios, que sugerem piores sintomas de ansiedade em pacientes com sintomas de compressão lombar mais intensos. Simultaneamente, a correlação positiva entre ganho de funcionalidade e ansiedade no momento pré-operatório demonstra que pacientes com mais sintomas de ansiedade tiveram benefício maior da cirurgia em questões de funcionalidade.

As principais limitações deste estudo são: casuística reduzida e limitada apenas a um centro hospitalar privado, podendo não representar a realidade de outros serviços, e a utilização do questionário HADS para rastreamento de ansiedade, o qual pode ser considerado limitado por falta de avaliação presencial de psicólogo. Devido à ausência da avaliação de comorbidades psiquiátricas nos pacientes bem como do uso de medicamentos, não foi possível avaliar possíveis efeitos entre comorbidades psiquiátricas, uso de medicamentos e o desenvolvimento dos sintomas de ansiedade nos pacientes após a cirurgia. Porém, a consistência dos dados sugere que a presença de dor neuropática e disfunção em indivíduos com compressão neurológica lombar promoveu o aparecimento de sintomas de ansiedade. Os fatores que corroboram com esta hipótese são: correlação inicial entre disfunção e ansiedade, seguidos por queda de ambos devido ao efeito terapêutico da cirurgia sobre a disfunção, reduzindo consequentemente a ansiedade.

## Conclusões

Cirurgia de descompressão lombar minimamente invasiva promoveu melhora clínica e funcional, não sendo afetada por sintomas pré-operatórios de ansiedade. Esses, por sua vez, melhoraram 6 meses após a cirurgia.
